# Modulation instability analysis and deriving soliton solutions of new nonlocal Lakshmanan–Porserzian–Daniel equation

**DOI:** 10.1038/s41598-025-21579-1

**Published:** 2025-11-10

**Authors:** Wafaa B. Rabie, W. Abbas, M. Elsaid Ramadan, Hamdy M. Ahmed

**Affiliations:** 1 Department of Mathematics, Faculty of Science, Luxor University, Taiba, Luxor, Egypt; 2 Basic and Applied Science Department, College of Engineering and Technology, Arab Academy for Science,Technology and Maritime Transport, Cairo, Egypt; 3 Department of Mathematics, Faculty of Science, Islamic University of Madinah, Medina, Saudi Arabia; 4 Department of Physics and Engineering Mathematics, Higher Institute of Engineering, El-Shorouk Academy, El-Shorouk City, Cairo Egypt

**Keywords:** Nonlocal LPD model, Wave propagation in complex media, Extended F-expansion method, Soliton, Stability analysis., Mathematics and computing, Optics and photonics, Physics

## Abstract

This study presents a comprehensive analytical exploration of the coupled nonlocal Lakshmanan-Porsezian-Daniel (LPD) equation, a seminal model for describing wave propagation in highly nonlocal nonlinear media. By employing the powerful extended F-expansion technique, we derive a rich spectrum of exact analytical solutions. These include bright, dark, and singular solitons, alongside singular periodic, periodic, Jacobi elliptic, exponential, hyperbolic, and Weierstrass elliptic wave solutions. The diversity of these solutions elucidates the profound and intricate interplay between strong nonlocality and nonlinearity in governing wave formation and evolution. Furthermore, we perform a detailed linear stability analysis to investigate the modulation instability (MI) gain spectrum within the system. This analysis identifies the critical parameters—most notably the degree of nonlocality and coupling strength—that dictate the stability regimes and the dynamic evolution of the solitons. Our analytical findings are vividly complemented by graphical representations that illustrate the distinctive structures of the obtained solutions and the precise conditions for the onset of MI. This research provides crucial insights into the robust propagation of localized waves in integrable nonlocal systems, with direct potential applications in pioneering fields such as nonlinear optics, Bose-Einstein condensates, and photonic lattice design, where precise control over wave dynamics is paramount.

## Introduction

Numerous physical phenomena, including those in solid mechanics, fluid dynamics, plasma physics, chemical processes, and atmospheric science, are represented by nonlinear partial differential equations (NPDEs). By applying various analytical techniques, it is possible to obtain exact solutions to these NPDEs^1–6^. Recent advancements in analytical methods, including Lie symmetry analysis^7^, bifurcation theory^8^, and stochastic analysis^9^, have significantly expanded our ability to solve complex nonlinear models across various scientific domains. In the past two decades, there have been numerous successful applications of nonlinear optical solitons in various fields of science and engineering^10–15^. The study of solitons has become a cornerstone in the examination of nonlinear wave phenomena across multiple scientific disciplines, ranging from fluid dynamics to optics. Solitons are unique in that they maintain their shape while propagating at constant speeds over long distances due to a precise balance between nonlinearity and dispersion^10^.

Localized pulses of light with a unique intensity, width, and shape are called optical solitons. These powerful beams retain their shape while propagating in material because their spreading due to three nonlinear effects can exactly balance each other. The first of these effects involves a direct distortion of the pulse, which usually leads to broadening; the second is self-phase modulation, where the phase acquired by the pulse is determined by its intensity; and the third is the Kerr-nonlinearity-induced change in the refractive index of the propagation medium that is either self-focusing or self-defocusing, further amplifying or attenuating the pulse itself. In optical contexts, the stability and propagation of these waveforms are crucial for advancements in telecommunications and data transfer technologies. Researchers have dedicated significant efforts to unpack the complexities of soliton dynamics, particularly in the presence of modulation instability (MI), which can lead to a marked change in the behavior of wave packets^11^. Among the models examining soliton interactions, the Lakshmanan-Porserzian-Daniel (LPD) system stands out due to its incorporation of nonlocal interactions, allowing for a rich tapestry of solitonic structures, including breather solutions and rogue waves^12^. Introduced to describe magnetic interactions in nonlinear systems, the LPD model has found applications in optical fiber analysis, where both the spatial and temporal dispersion of waves is of paramount importance^13^. Modulation instability plays a pivotal role in the evolution of solitons within nonlinear systems. MI occurs when a stationary solution becomes unstable under perturbation, leading to the formation of new wave structures, often characterized by significant amplitude fluctuations. It is particularly noteworthy in optical systems where external conditions can precipitate such instabilities, potentially resulting in phenomena such as soliton explosions^14^. Understanding and controlling MI is therefore of critical importance, particularly in contexts like optical communications, where signal integrity relies on the predictable behavior of solitonic waves^15^. Inspecting the dynamics of optical solitons through optical fibers is essential in the development of telecommunications industry (see^16–28^). In this research, we delve into the dynamics of the coupled nonlocal LPD system by employing the Extended F-Expansion technique, which proves advantageous in deriving exact solutions for a variety of nonlinear partial differential equations. This analytical method has emerged as a powerful tool for exploring and characterizing complex solitonic solutions, offering insight into their stability and interaction properties^29^. Recent advances in soliton research have further expanded our understanding of nonlinear wave dynamics in optical fibers. Various studies have explored shape-changing solitons, chirped dark solitons, and W-shaped solitons in different nonlinear Schrödinger equation frameworks^30–32^. Additionally, investigations into dark and singular solitons using extended rational methods have provided new insights into soliton behavior in nonlinear optical systems^33^. The exploration of W and M shaped solitons in eighth-order nonlinear Schrödinger equations has revealed complex soliton structures^34^, while studies on inhomogeneous vector optical rogue waves have demonstrated deformation effects in coupled cubic-quintic nonlinear Schrödinger equations^35^. Research on instabilities and solitons in systems with spatio-temporal dispersions has enhanced our understanding of modulational instability phenomena^36^. Complementary studies on energy localization in tubulin systems^37^ and modulational instability in spin-orbit-coupled Bose-Einstein condensates^38^ have provided additional perspectives on nonlinear wave dynamics. Furthermore, investigations into the impact of fourth-order dispersion on modulational instability spectra have contributed to our understanding of wave propagation in glass fibers with saturable nonlinearity^39^. In our study, we treat the coupled system described by the new nonlocal Lakshmanan-Porserzian-Daniel equations as^40^:

$$\begin{aligned} iG_t+G_{xxxx}+4\left( \left| G\right| ^2+\left| Q\right| ^2\right) G_{xx}+2G_x\left[ 2\left( G_x G^*+Q_x Q^*\right) +\left( \left| G\right| ^2+\left| Q\right| ^2 \right) _x\right] \end{aligned}$$

1 $$\begin{aligned} +2G\left[ 2G_{xx}G^* +G_x G^*_x+G G^*_{xx}+2Q_{xx}Q^* +Q_x Q^*_x+Q Q^*_{xx}+3\left( \left| G\right| ^2+\left| Q\right| ^2 \right) ^2 \right] =0, \end{aligned}$$

$$\begin{aligned} iQ_t+Q_{xxxx}+4\left( \left| G\right| ^2+\left| Q\right| ^2\right) Q_{xx}+2Q_x\left[ 2\left( G_x G^*+Q_x Q^*\right) +\left( \left| G\right| ^2+\left| Q\right| ^2 \right) _x \right] \end{aligned}$$

2 $$\begin{aligned} +2Q\left[ 2G_{xx}G^* +G_x G^*_x+G G^*_{xx}+2Q_{xx}Q^* +Q_x Q^*_x+Q Q^*_{xx}+3\left( \left| G\right| ^2+\left| Q\right| ^2 \right) ^2 \right] =0, \end{aligned}$$

where *G*(*x*, *t*),  *Q*(*x*, *t*) are the wave profiles and *t*,  *x* are time and space variables.

In the current study, the extended F-expansion technique is employed to derive multiple exact solutions–including bright, dark, and singular solitons, as well as singular periodic, periodic, Jacobi elliptic, exponential, hyperbolic, and Weierstrass elliptic solutions–for the proposed coupled nonlocal Lakshmanan–Porsezian–Daniel (LPD) model. This work addresses a significant research gap in the current literature, where comprehensive studies on exact soliton solutions for the coupled nonlocal LPD system remain limited. The novelty of this study lies in the systematic exploration of both coherent structures (solitons) and modulation instability in the same framework, offering a unified view of nonlinear wave dynamics in nonlocal media. Furthermore, we extend the application of the extended F-expansion method to a coupled system with nonlocal interactions, yielding a richer variety of solutions than previously reported. These findings provide new insights into the propagation and stability of optical solitons in settings with spatially nonlocal nonlinearities, which have potential implications for advanced telecommunications and optical computing. To illustrate the physical characteristics of the obtained solutions, three-dimensional and two-dimensional graphical representations of selected results are presented.

The paper is structured into the following sections: “Summary method” presents the proposed method in detail. It provides a comprehensive framework for understanding the methodologies used throughout the study, focusing on the technical aspects and rationale behind the approach used. The results derived from the application of the proposed method are discussed in “Soliton dynamics for LPD system”. It explains the results and their implications, supported by the data and analyses conducted during the research. Section “Soliton dynamics for LPD system” discusses the modulation instability analysis of the model. Section “Modulation instability” uses both 3D simulations and 2D graphs to illustrate the different dynamic wave patterns across different insulation solutions. This visual representation enhances the readers’ understanding of the results and their practical applications. The final section concludes the work. It summarizes the main findings, discusses their significance and potential applications, and suggests future research directions.

##  Summary method

The F-Expansion method is a powerful mathematical technique for deriving exact solutions to NLPDEs^28^.

***Step 1:*** Assume that an NLPDE is displayed as follows:

3 $$\begin{aligned} \mathfrak {R} \left( G, G_t, G_x, G_{xx},...\right) = 0. \end{aligned}$$

***Step 2:*** Eq. (3) is transformed into an ODE using the traveling wave transformation shown below:

4 $$\begin{aligned} G (x,t)= P~[\vartheta ],~~\vartheta =x+\varphi ~ t, \end{aligned}$$

where $\varphi$ represents the soliton’s propagation speed. Thus, Eq.(3) can be expressed as follows:

5 $$\begin{aligned} \mathfrak {K} (P, P^\prime , P^{\prime \prime }, \ldots ) = 0. \end{aligned}$$

***Step 3:*** The general solution is expressed as a polynomial in terms of a function $\mu (\vartheta )$ as follows:

6 $$\begin{aligned} P\left[ \vartheta \right] =\eta _{0}+\sum _{i =1}^N \left[ \eta _{i}\ \mu ^i (\vartheta )+ \lambda _{i}\ \mu ^{-i}(\vartheta ) \right] , \end{aligned}$$

where $\eta _{i}$ and $\lambda _{i}~ (i=1,2,...,N)$ represent constants in the solution equation that need to be determined, while the function $\mu (\vartheta )$  achieves the following constrain:

7 $$\begin{aligned} \mu ^\prime (\vartheta ) =\alpha ~ \sqrt{\tau _0 +\tau _1~\mu ~+~\tau _2~\mu ^2 ~+~\tau _3~\mu ^3 ~+~\tau _4~\mu ^4},~~~ \end{aligned}$$

and $$~\alpha =\pm 1$$.

***Step 4:*** In order to assess the positive integer *N*, the balancing principle (BP) is employed to Eq.(5).

***Step 5:*** Substituting Eqs. (6) and (7) into Eq. (5) generates a polynomial in terms of $\mu$. Setting the coefficients of $\mu$ to zero through algebraic polynomial operations results in a nonlinear system of algebraic equations. This system can be solved using various computational tools, such as Wolfram Mathematica, leading to multiple exact solutions for Eq. (3).

## Soliton dynamics for LPD system

To generate the exact solutions for Eqs.(1) and (2), we apply the transformation displayed below:

8 $$\begin{aligned} G (x,t)= P_1~[\vartheta ]~ e^{i (- x \kappa +\zeta + t \omega )}, \end{aligned}$$

9 $$\begin{aligned} Q (x,t)= P_2~[\vartheta ]~ e^{i (- x \kappa +\zeta + t \omega )}, \end{aligned}$$

where

10 $$\begin{aligned} \vartheta = x+\varphi ~t, \end{aligned}$$

Here, $P_j$ ( where $j=1,2$ ) denotes the amplitude parameters, $\varphi$ stands for the soliton’s propagation speed, while $\omega$ and $\kappa$ correspond to its angular frequency and wave number, respectively. The term $\zeta$ represents the phase of the wave. By inserting Eqs. (8), (9), and (10) into Eqs. (1) and (2), and then separating the resulting expression into their real and imaginary components, we arrive at the following system:

$$\begin{aligned} P_j^{(4)}+\left( \kappa ^4-\omega \right) P_j-12\kappa ^2 P_j^3+6P_j^5-12\kappa ^2 P_jP_{\tilde{j}}^2+12P_{\tilde{j}}^2P_j^3+6P_{\tilde{j}}^4P_j \end{aligned}$$

11 $$\begin{aligned} +10P_j(P^{\prime }_j)^2+8P_{\tilde{j}}P^{\prime }_jP_{\tilde{j}}^{\prime }+2P_jP_{\tilde{j}}^{\prime ^2}-6\kappa ^2P_j^{\prime \prime }+10P_j^2P_j^{\prime \prime }+4P_{\tilde{j}}^2P_j^{\prime \prime }+6P_jP_{\tilde{j}}P_{\tilde{j}}^{\prime \prime }=0, \end{aligned}$$

12 $$\begin{aligned} \left( 4\kappa ^3+\varphi \right) P_{\tilde{j}}^{\prime }-24\kappa P^2_jP_j^{\prime }-12\kappa P_{\tilde{j}}^2 P^{\prime }_j-12\kappa P_j P_{\tilde{j}}P_{\tilde{j}}^{\prime }-4\kappa P^{(3)}_j=0. \end{aligned}$$

We presume that:

13 $$\begin{aligned} P_{\tilde{j}}=h~P_j, \end{aligned}$$

with the condition $h \ne 0,~1$.

Accordingly, Eqs. (11) and (12) take the form:

14 $$\begin{aligned}&\nonumber P_j^{(4)} + (\kappa ^4 - \omega )P_j - 12\kappa ^2 P_j^3 + 6P_j^5 - 12 \ h^2\ \kappa ^2 \ P_j^3+ 12h^2 \ P_j^5 + 6h^4\ P_j^5 + 10P_j(P^{\prime }_j)^2 + 8h^2 \ P_j \ (P^{\prime }_j)^2 \\&+ 2h^2 P_j \ (P^{\prime }_j)^2 - 6\ \kappa ^2 P_j^{\prime \prime } + 10 \ P_j^2\ P_j^{\prime \prime } + 4h^2 P_j^2 \ P_j^{\prime \prime } + 6h^2\ P_j^2 \ P_j^{\prime \prime } = 0, \end{aligned}$$

15 $$\begin{aligned}&h(4\kappa ^3 + \varphi )P^{\prime }_j - 24\kappa P^2_j P^{\prime }_j - 12h^2\kappa P_j^2 P^{\prime }_j - 12h^2\kappa P_j^2 P^{\prime }_j - 4\kappa P^{(3)}_j = 0 \end{aligned}$$

Therefore, the above equations can be formulated as:

$$\begin{aligned} P_j^{(4)}+10 \left( h^2+1\right) P_j^2 P_j''-6 \kappa ^2 P_j''+10 \left( h^2+1\right) P_j \left( P_j'\right) ^2+\left( \kappa ^4-\omega \right) \ P_j+\left( -12 h^2 \kappa ^2-12 \kappa ^2\right) P_j^3+ \end{aligned}$$

16 $$\begin{aligned} \left( 6 h^4+12 h^2+6\right) P_j^5=0, \end{aligned}$$

17 $$\begin{aligned} -\left( 4 \kappa ^3+\rho \right) P_j'+6 \kappa \left( 1+h^2 \right) P^2_j P^{\prime }_j+\kappa P^{(3)}_j=0. \end{aligned}$$

By performing integration of Eq. (17) with respect to $\vartheta$ and assuming the constant of integration is zero, we obtain:

18 $$\begin{aligned} -\left( 4 \kappa ^3+\rho \right) P_j+2 \kappa \left( 1+h^2 \right) P^3_j+\kappa P_j''=0. \end{aligned}$$

Through Eq.(18), then Eq.(16) can be formulated as:

19 $$\begin{aligned} & \kappa P_j^{(4)}+10 \left( h^2+1\right) \kappa P_j \left( P_j'\right) {}^2-14 \left( h^2+1\right) ^2 \kappa P_j^5+10 \left( h^2+1\right) \left( 4 \kappa ^3+\rho \right) P_j^3\nonumber \\ & \quad -\kappa \left( 23 \kappa ^4+6 \kappa \rho +\omega \right) P_j =0. \end{aligned}$$

By applying the **Balancing Principle (BP)** between the highest order derivative $P_j^{(4)}$ and the highest nonlinear term $P_j^5$ in Eq.(17), we find $m+4=5m$, then $m=1$. The resulting exact solution of Eq. (17) takes the form:

20 $$\begin{aligned} P[\vartheta ]=\eta _0+\eta _1 ~\mu [\vartheta ]+\frac{\lambda _1}{\mu [\vartheta ]}. \end{aligned}$$

Inserting Eq. (20) into Eq. (19) while incorporating Eq. (7), and equating the coefficients of identical powers to zero, results in a system of nonlinear algebraic equations. This system is formed by grouping terms with the same powers and setting them equal to zero. The resulting equations can be solved using Wolfram Mathematica to provide the following results:

**Case 1**:   $$\tau _1 =\tau _0 =\tau _3 =0$$,

$$(1.1) \quad \eta _0=0,\ \eta _1=\sqrt{-\frac{\tau _4}{h^2+1}},\ \lambda _1=0,\ \omega =-7 \kappa ^4+\frac{\rho ^2}{\kappa ^2}+2 \kappa \rho ,\ \tau _2=\frac{4 \kappa ^3+\rho }{ \kappa }.$$

$$(1.2) \quad \eta _0=0,\ \eta _1=2 \sqrt{\frac{3 \tau _4}{7 \left( h^2+1\right) }},\ \lambda _1=0,\ \omega =-\frac{3311 \kappa ^6+726 \kappa ^3 \rho -36 \rho ^2}{169 \kappa ^2},\ \tau _2=-\frac{6 \left( 4 \kappa ^3+\rho \right) }{13 \kappa }$$

Through the case (1.1), we can get all possible results as follows: 

(1.1, 1) When $\rho =-4 \kappa ^3,~and~\tau _4=-1$, we get bright solitons as:

21 $$\begin{aligned} G _{1.1}=\sqrt{\frac{1}{h^2+1}} \ {{\,\textrm{sech}\,}}\left( x-4 \kappa ^3 t\right) ~ e^{i (- x \kappa +\zeta + t \omega )}, \end{aligned}$$

22 $$\begin{aligned} Q _{1.1}=h\ \sqrt{\frac{1}{h^2+1}} \ {{\,\textrm{sech}\,}}\left( x-4 \kappa ^3 t\right) ~ e^{i (- x \kappa +\zeta + t \omega )}. \end{aligned}$$

Through the case (1.2), we can get all possible results as follows:

(1.2, 1) For $\rho =\frac{13 \kappa }{6}-4 \kappa ^3,~and~\tau _4=1$, the singular periodic solutions take the forms:

23 $$\begin{aligned} G _{1.2,1}=2 \ \sqrt{\frac{3}{7 \left( h^2+1\right) }} \csc \left[ x+\left( \frac{13 \kappa }{6}-4 \kappa ^3\right) t\right] ~ e^{i (- x \kappa +\zeta + t \omega )}, \end{aligned}$$

24 $$\begin{aligned} Q _{1.2, 1}= 2 \ h\ \sqrt{\frac{3}{7 \left( h^2+1\right) }} \csc \left[ x+\left( \frac{13 \kappa }{6}-4 \kappa ^3\right) t\right] ~e^{i (- x \kappa +\zeta + t \omega )}, \end{aligned}$$

or

25 $$\begin{aligned} G _{1.2,2}=2 \ \sqrt{\frac{3}{7 \left( h^2+1\right) }} \sec \left[ x+\left( \frac{13 \kappa }{6}-4 \kappa ^3\right) t\right] ~ e^{i (- x \kappa +\zeta + t \omega )}, \end{aligned}$$

26 $$\begin{aligned} Q _{1.2, 2}= 2 \ h\ \sqrt{\frac{3}{7 \left( h^2+1\right) }} \sec \left[ x+\left( \frac{13 \kappa }{6}-4 \kappa ^3\right) t\right] ~e^{i (- x \kappa +\zeta + t \omega )}, \end{aligned}$$

(1.2, 2) For $\rho =-\frac{13 \kappa }{6}-4 \kappa ^3,~and~\tau _4=1$, the singular solitons of Eqs.(1) and (2) are given as:

27 $$\begin{aligned} G _{1.2,3}=2 \ \sqrt{\frac{3}{7 \left( h^2+1\right) }} {{\,\textrm{csch}\,}}\left[ x-\left( \frac{13 \kappa }{6}+4 \kappa ^3\right) t\right] ~ e^{i (- x \kappa +\zeta + t \omega )}, \end{aligned}$$

28 $$\begin{aligned} Q _{1.2, 3}= 2 \ h\ \sqrt{\frac{3}{7 \left( h^2+1\right) }} {{\,\textrm{csch}\,}}\left[ x-\left( \frac{13 \kappa }{6}+4 \kappa ^3\right) t\right] ~e^{i (- x \kappa +\zeta + t \omega )}. \end{aligned}$$

(1.2, 3) For $\tau _4=1$ and either $\rho =-(\frac{13 \kappa }{6}+4 \kappa ^3),~ \text {or}~\rho =(\frac{13 \kappa }{6}-4 \kappa ^3)$, we get hyperbolic or periodic solutions as shown:

29 $$\begin{aligned} G _{1.2,4}=2 \ \sqrt{\frac{3}{7 \left( h^2+1\right) }} \sinh \left[ x-\left( \frac{13 \kappa }{6}+4 \kappa ^3\right) t\right] ~ e^{i (- x \kappa +\zeta + t \omega )}, \end{aligned}$$

30 $$\begin{aligned} Q _{1.2, 4}= 2 \ h\ \sqrt{\frac{3}{7 \left( h^2+1\right) }} \sinh \left[ x-\left( \frac{13 \kappa }{6}+4 \kappa ^3\right) t\right] ~e^{i (- x \kappa +\zeta + t \omega )}, \end{aligned}$$

or

31 $$\begin{aligned} G _{1.2,5}=2 \ \sqrt{\frac{3}{7 \left( h^2+1\right) }} \sin \left[ x+\left( \frac{13 \kappa }{6}-4 \kappa ^3\right) t\right] ~ e^{i (- x \kappa +\zeta + t \omega )}, \end{aligned}$$

32 $$\begin{aligned} Q _{1.2, 5}= 2 \ h\ \sqrt{\frac{3}{7 \left( h^2+1\right) }} \sin \left[ x+\left( \frac{13 \kappa }{6}-4 \kappa ^3\right) t\right] ~e^{i (- x \kappa +\zeta + t \omega )}. \end{aligned}$$

(1.2, 4) When $\tau _2=0$ and $\tau _4>0$ hence, we derive rational solutions as:

33 $$\begin{aligned} G _{1.2,6}=-\frac{2 \alpha \sqrt{3} }{ (\rho t+x) \ \sqrt{7 \left( h^2+1\right) }}~ e^{i (- x \kappa +\zeta + t \omega )}, \end{aligned}$$

34 $$\begin{aligned} Q _{1.2, 5}= -\frac{2 \alpha \ h\ \sqrt{3} }{ (\rho t+x) \ \sqrt{7 \left( h^2+1\right) }}~ e^{i (- x \kappa +\zeta + t \omega )}. \end{aligned}$$

**Case 2:**  $$\tau _1 =\tau _3 =0$$.

$$(2.1) \quad \eta _0=0,\ \eta _1=\sqrt{-\frac{\tau _4}{h^2+1}},\ \lambda _1=0,\ \tau _0=\frac{7 \kappa ^6-2 \kappa ^3 \rho +\kappa ^2 \omega -\rho ^2}{2 \kappa ^2 \tau _4},\ \tau _2=\frac{4 \kappa ^3+\rho }{\kappa }$$

$$(2.2) \quad \eta _0=0,\ \eta _1=2 \sqrt{\frac{3 \tau _4}{7 \left( h^2+1\right) }},\ \lambda _1=0,\ \tau _0=\frac{7 \left( 3311 \kappa ^6+726 \kappa ^3 \rho +169 \kappa ^2 \omega -36 \rho ^2\right) }{34476 \kappa ^2 \tau _4},\ \tau _2=-\frac{6 \left( 4 \kappa ^3+\rho \right) }{13 \kappa }$$

$$(2.3) \quad \eta _0=0,\ \eta _1=0,\ \lambda _1=\pm \sqrt{-\frac{7 \kappa ^6-2 \kappa ^3 \rho +\kappa ^2 \omega -\rho ^2}{2 \left( \left( h^2+1\right) \kappa ^2 \tau _4\right) }},\ \tau _0=\frac{7 \kappa ^6-2 \kappa ^3 \rho +\kappa ^2 \omega -\rho ^2}{2 \alpha ^4 \kappa ^2 \tau _4},\ \tau _2=\frac{4 \kappa ^3+\rho }{\alpha ^2 \kappa }$$

Through the case (2.1), we can get all possible results as follows:

(2.1, 1): When $\tau _0=1-m^2, \ \tau _2=m^2-1, \ \tau _4=-m^2$ and $0<m\le 1$, we derive Jacobi elliptic function solutions as:

35 $$\begin{aligned} G _{2.1, 1}=\sqrt{\frac{m^2}{h^2+1}} {{\,\textrm{cn}\,}}\left[ x+\left( \alpha ^2 \kappa \left( m^2-1\right) -4 \kappa ^3\right) t \right] ~ e^{i (- x \kappa +\zeta + t \omega )}, \end{aligned}$$

36 $$\begin{aligned} Q _{2.1, 1}=h\ \sqrt{\frac{m^2}{h^2+1}} {{\,\textrm{cn}\,}}\left[ x+\left( \alpha ^2 \kappa \left( m^2-1\right) -4 \kappa ^3\right) t \right] ~ ~ e^{i (- x \kappa +\zeta + t \omega )}. \end{aligned}$$

When the parameter $m= 1$, Eqs. (35) and (36) yields the following bright soliton solutions:

37 $$\begin{aligned} G _{2.1, 2}=\sqrt{\frac{1}{h^2+1}} \ {{\,\textrm{sech}\,}}\left( x-4 \kappa ^3 t\right) ~ e^{i (- x \kappa +\zeta + t \omega )}, \end{aligned}$$

38 $$\begin{aligned} Q _{2.1, 2}=h\ \sqrt{\frac{1}{h^2+1}} \ {{\,\textrm{sech}\,}}\left( x-4 \kappa ^3 t\right) ~ e^{i (- x \kappa +\zeta + t \omega )}. \end{aligned}$$

(2.1, 2): When $\tau _0= -1+m^2, \ \tau _2=2-m^2, \ \tau _4=-1$ and $m \in [0,1]$, we derive Jacobi elliptic function solutions as:

39 $$\begin{aligned} G _{2.1, 3}=\sqrt{\frac{1}{h^2+1}} {{\,\textrm{dn}\,}}\left[ x-\left( \alpha ^2 \kappa \left( -2+m^2\right) +4 \kappa ^3\right) t \right] ~ e^{i (- x \kappa +\zeta + t \omega )}, \end{aligned}$$

40 $$\begin{aligned} Q _{2.1, 3}=h\ \sqrt{\frac{1}{h^2+1}} {{\,\textrm{dn}\,}}\left[ x-\left( \alpha ^2 \kappa \left( -2+m^2\right) +4 \kappa ^3\right) t \right] ~ ~ e^{i (- x \kappa +\zeta + t \omega )}. \end{aligned}$$

When the parameter $m= 1$, Eqs. (39) and (40) yields the following bright soliton solutions:

41 $$\begin{aligned} G _{2.1,4}=\sqrt{\frac{1}{h^2+1}} \ {{\,\textrm{sech}\,}}\left( x+\left( \alpha ^2-4 \kappa ^2\right) \ \kappa t\right) ~ e^{i (- x \kappa +\zeta + t \omega )}, \end{aligned}$$

42 $$\begin{aligned} Q _{2.1, 4}=h\ \sqrt{\frac{1}{h^2+1}} \ {{\,\textrm{sech}\,}}\left( x+\left( \alpha ^2-4 \kappa ^2\right) \ \kappa t\right) ~ e^{i (- x \kappa +\zeta + t \omega )}. \end{aligned}$$

Through the case (2.2), we can get all possible results as follows:

(2.2, 1): When $\tau _2<0, \ \tau _4>0$ and $\tau _0=\frac{\tau _2^2}{4 \tau _4}$, we get dark solitons as:

43 $$\begin{aligned} G _{2.2, 1}=2 \ \sqrt{-\frac{3 \ \tau _2}{14 \left( h^2+1\right) }} \ \tanh \left[ (\rho \ t +x) \ \sqrt{-\frac{\tau _2}{2}} \right] ~ e^{i (- x \kappa +\zeta + t \omega )}, \end{aligned}$$

44 $$\begin{aligned} Q _{2.2, 1}=2\ h\ \ \sqrt{-\frac{3 \ \tau _2}{14 \left( h^2+1\right) }} \ \tanh \left[ (\rho \ t +x) \ \sqrt{-\frac{\tau _2}{2}} \right] ~ e^{i (- x \kappa +\zeta + t \omega )}. \end{aligned}$$

(2.2, 2): When $\tau _2>0, \ \tau _4>0$ and $\tau _0=\frac{\tau _2^2}{4 \tau _4}$, we derive singular periodic solutions as:

45 $$\begin{aligned} G _{2.2, 2}=2 \ \sqrt{\frac{3 \ \tau _2}{14 \left( h^2+1\right) }} \ \tan \left[ (\rho \ t +x) \ \sqrt{\frac{\tau _2}{2}} \right] ~ e^{i (- x \kappa +\zeta + t \omega )}, \end{aligned}$$

46 $$\begin{aligned} Q _{2.2, 2}=2\ h\ \ \sqrt{\frac{3 \ \tau _2}{14 \left( h^2+1\right) }} \ \tan \left[ (\rho \ t +x) \ \sqrt{\frac{\tau _2}{2}} \right] ~ e^{i (- x \kappa +\zeta + t \omega )}. \end{aligned}$$

(2.2, 3): When $\tau _2<0, \ \tau _4>0$, $\tau _0=\frac{m^2 \tau _2^2}{\left( m^2+1\right) ^2 \tau _4}$ and $0<m\le 1$, we derive Jacobi elliptic function solutions as:

47 $$\begin{aligned} G _{2.2, 3}=2 \ \sqrt{-\frac{3\ m^2 \ \tau _2}{7 \left( h^2+1\right) \left( m^2+1\right) }} \ {{\,\textrm{sn}\,}}\left[ (\rho \ t +x) \ \sqrt{-\frac{\tau _2}{m^2+1}}\ \sqrt{\frac{\tau _2}{2}} \right] ~ e^{i (- x \kappa +\zeta + t \omega )}, \end{aligned}$$

48 $$\begin{aligned} Q _{2.2, 3}=2\ h\ \ \sqrt{-\frac{3\ m^2 \ \tau _2}{7 \left( h^2+1\right) \left( m^2+1\right) }} \ {{\,\textrm{sn}\,}}\left[ (\rho \ t +x) \ \sqrt{-\frac{\tau _2}{m^2+1}}\ \sqrt{\frac{\tau _2}{2}} \right] ~ e^{i (- x \kappa +\zeta + t \omega )}. \end{aligned}$$

When the parameter $m= 1$, Eqs. (47) and (48) yields the following dark soliton solutions:

49 $$\begin{aligned} G _{2.2,4}=2\ \sqrt{\frac{-3\ \tau _2}{14(h^2+1)}} \ \tanh \left[ (\rho \ t +x) \ \sqrt{-\frac{\tau _2}{2}}\right] ~ e^{i (- x \kappa +\zeta + t \omega )}, \end{aligned}$$

50 $$\begin{aligned} Q _{2.2, 4}=2\ h\ \sqrt{\frac{-3\ \tau _2}{14(h^2+1)}} \ \tanh \left[ (\rho \ t +x) \ \sqrt{-\frac{\tau _2}{2}}\right] ~ e^{i (- x \kappa +\zeta + t \omega )}. \end{aligned}$$

Through the case (2.3), we can get all possible results as follows:

(2.3, 1): When $\tau _2<0, \ \tau _4>0$, $\tau _0=\frac{\tau _2^2}{4 \tau _4}$ and $7 \kappa ^6-2 \kappa ^3 \rho +\kappa ^2 \omega -\rho ^2<0$, hence, we obtain singular soliton solutions of Eqs.(1) and (2) as shown:

51 $$\begin{aligned} G _{2.3, 1}=\pm \sqrt{\frac{7 \kappa ^6-2 \kappa ^3 \rho +\kappa ^2 \omega -\rho ^2}{\left( h^2+1\right) \kappa ^2 \tau _2}} \coth \left[ (\rho \ t +x) \ \sqrt{-\frac{\tau _2}{2}} \right] ~ e^{i (- x \kappa +\zeta + t \omega )}, \end{aligned}$$

52 $$\begin{aligned} Q _{2.3, 1}=\pm h \ \sqrt{\frac{7 \kappa ^6-2 \kappa ^3 \rho +\kappa ^2 \omega -\rho ^2}{\left( h^2+1\right) \kappa ^2 \tau _2}} \coth \left[ (\rho \ t +x) \ \sqrt{-\frac{\tau _2}{2}} \right] ~ e^{i (- x \kappa +\zeta + t \omega )}. \end{aligned}$$

(2.3, 2): When $\tau _2>0, \ \tau _4>0$, $\tau _0=\frac{\tau _2^2}{4 \tau _4}$ and $7 \kappa ^6-2 \kappa ^3 \rho +\kappa ^2 \omega -\rho ^2<0$, we derive singular periodic solutions as:

53 $$\begin{aligned} G _{2.3, 2}=\pm \sqrt{\frac{-(7 \kappa ^6-2 \kappa ^3 \rho +\kappa ^2 \omega -\rho ^2)}{\left( h^2+1\right) \kappa ^2 \tau _2}} \cot \left[ (\rho \ t +x) \ \sqrt{\frac{\tau _2}{2}} \right] ~ e^{i (- x \kappa +\zeta + t \omega )}, \end{aligned}$$

54 $$\begin{aligned} Q _{2.3, 2}=\pm \sqrt{\frac{-(7 \kappa ^6-2 \kappa ^3 \rho +\kappa ^2 \omega -\rho ^2)}{\left( h^2+1\right) \kappa ^2 \tau _2}} \cot \left[ (\rho \ t +x) \ \sqrt{\frac{\tau _2}{2}} \right] ~ e^{i (- x \kappa +\zeta + t \omega )}. \end{aligned}$$

(2.3, 3): When $\tau _2>0, \ \tau _4<0$, $\tau _0=\frac{m^2 \left( 1-m^2\right) \tau _2^2}{\left( 2 m^2-1\right) ^2 \tau _4}$, $\left( 2 m^2-1\right) \left( 7 \kappa ^6-2 \kappa ^3 \rho +\kappa ^2 \omega -\rho ^2\right) >0$ and $\frac{1}{\sqrt{2}}<m\le 1$, we get Jacobi elliptic function solutions as:

55 $$\begin{aligned} G _{2.3, 3}=\pm \sqrt{\frac{\left( 2 m^2-1\right) \left( 7 \kappa ^6-2 \kappa ^3 \rho +\kappa ^2 \omega -\rho ^2\right) }{\alpha ^2 \left( h^2+1\right) \kappa ^2 m^2 \tau _2}} \ {{\,\textrm{nc}\,}}\left[ (\rho \ t +x) \ \sqrt{\frac{\tau _2}{2 m^2-1}}\right] ~ e^{i (- x \kappa +\zeta + t \omega )}, \end{aligned}$$

56 $$\begin{aligned} Q _{2.3, 3}=\pm h \sqrt{\frac{\left( 2 m^2-1\right) \left( 7 \kappa ^6-2 \kappa ^3 \rho +\kappa ^2 \omega -\rho ^2\right) }{\alpha ^2 \left( h^2+1\right) \kappa ^2 m^2 \tau _2}} \ {{\,\textrm{nc}\,}}\left[ (\rho \ t +x) \ \sqrt{\frac{\tau _2}{2 m^2-1}}\right] ~ e^{i (- x \kappa +\zeta + t \omega )}. \end{aligned}$$

When the parameter $m= 1$, Eqs. (55) and (56) yields the following hyperbolic solutions:

57 $$\begin{aligned} G _{2.3,4}=\pm \sqrt{\frac{7 \kappa ^6-2 \kappa ^3 \rho +\kappa ^2 \omega -\rho ^2}{\alpha ^2 \left( h^2+1\right) \kappa ^2 \tau _2}}\ \cosh \left[ (\rho \ t +x) \ \sqrt{\tau _2}\right] ~ e^{i (- x \kappa +\zeta + t \omega )}, \end{aligned}$$

58 $$\begin{aligned} Q _{2.3, 4}=\pm h \sqrt{\frac{7 \kappa ^6-2 \kappa ^3 \rho +\kappa ^2 \omega -\rho ^2}{\alpha ^2 \left( h^2+1\right) \kappa ^2 \tau _2}}\ \cosh \left[ (\rho \ t +x) \ \sqrt{\tau _2}\right] ~e^{i (- x \kappa +\zeta + t \omega )}. \end{aligned}$$

**Case 3:** $\tau _2 =\tau _4 =0.$ $\eta _0=\frac{2 \sqrt{3 \left( 4 \kappa ^3+\rho \right) }}{\sqrt{91 \left( h^2+1\right) \kappa }}, \eta _1=0, \ \lambda _1=\frac{8 \tau _0 \sqrt{3 \left( 4 \kappa ^3+\rho \right) }}{\tau _1 \sqrt{91 \left( h^2+1\right) \kappa }}, \alpha = -\frac{4 \sqrt{\tau _0 \left( 4 \kappa ^3+\rho \right) }}{\sqrt{13 \kappa } \tau _1}, \omega =\frac{-6857 \kappa ^6+3078 \kappa ^3 \rho +1272 \rho ^2}{1183 \kappa ^2}, \tau _3=-\frac{\tau _1^3}{8 \tau _0^2}$. Through this case, all attainable outcomes are derived as follows:

When $\tau _1<0,\ \tau _0<0,\text {and} \ \kappa \left( 4 \kappa ^3+\rho \right) >0$, hence, we obtain Weierstrass elliptic function solutions as:

59 $$\begin{aligned} G _{3.1}=\pm \frac{\sqrt{3 \left( 4 \kappa ^3+\rho \right) }}{\sqrt{91 \left( h^2+1\right) \kappa }}~ \left[ \frac{2 \left[ 4 \tau _0+\tau _1 \wp \left( \sqrt{\frac{\tau _3}{4}} (\rho t+x),-\frac{4 \tau _1}{\tau _3},-\frac{4 \tau _0}{\tau _3}\right) \right] }{\tau _1 \sqrt{\left( h^2+1\right) \kappa } \ \wp \left( \sqrt{\frac{\tau _3}{4}} (\rho t+x),-\frac{4 \tau _1}{\tau _3},-\frac{4 \tau _0}{\tau _3}\right) }\right] e^{i (- x \kappa +\zeta + t \omega )}, \end{aligned}$$

60 $$\begin{aligned} Q _{3.1}=\pm h \frac{\sqrt{3 \left( 4 \kappa ^3+\rho \right) }}{\sqrt{91 \left( h^2+1\right) \kappa }}~ \left[ \frac{2 \left[ 4 \tau _0+\tau _1 \wp \left( \sqrt{\frac{\tau _3}{4}} (\rho t+x),-\frac{4 \tau _1}{\tau _3},-\frac{4 \tau _0}{\tau _3}\right) \right] }{\tau _1 \sqrt{\left( h^2+1\right) \kappa } \ \wp \left( \sqrt{\frac{\tau _3}{4}} (\rho t+x),-\frac{4 \tau _1}{\tau _3},-\frac{4 \tau _0}{\tau _3}\right) }\right] ~ e^{i (- x \kappa +\zeta + t \omega )}. \end{aligned}$$

**Case 4:** $$\tau _3 =\tau _4 =0.$$ $$\eta _0=\frac{6 \sqrt{4 \kappa ^3+\rho }}{\sqrt{91 \left( h^2+1\right) \kappa }},\ \eta _1=0,\ \lambda _1=\frac{ \kappa \ \tau _1 \ \sqrt{13}}{2 \sqrt{7 \ \kappa \ \left( h^2+1\right) \left( 4 \kappa ^3+\rho \right) }},\ \omega =\frac{6199 \kappa ^6+9606 \kappa ^3 \rho +2088 \rho ^2}{1183 \kappa ^2},\ \tau _0=\frac{13 \ \kappa \ \tau _1^2}{48 \left( 4 \kappa ^3+\rho \right) }, \tau _2=\frac{12 \left( 4 \kappa ^3+\rho \right) }{13\ \alpha ^2 \ \kappa }$$.

In this scenario, all attainable outcomes are derived as follows: When $\tau _2>0\ \text {and} \ \kappa \left( 4 \kappa ^3+\rho \right) >0$, we obtain exponential solutions as:

61 $$\begin{aligned} G _{4.1}=- \frac{\sqrt{\left( 4 \kappa ^3+\rho \right) }}{\sqrt{91 \left( h^2+1\right) \kappa }}~ \left[ \frac{6 \left( 2 \ \tau _2\ e^{[ (\rho \ t +x)\ \sqrt{\tau _2}]}+\tau _1 \left( 2 \ \tau _2-1\right) \right) }{\tau _1-2 \tau _2 \ e^{[(x+\rho \ t)\ \sqrt{\tau _2}] }}\right] e^{i (- x \kappa +\zeta + t \omega )}, \end{aligned}$$

62 $$\begin{aligned} Q _{4.1}=- h\ \frac{\sqrt{\left( 4 \kappa ^3+\rho \right) }}{\sqrt{91 \left( h^2+1\right) \kappa }}~ \left[ \frac{6 \left( 2 \ \tau _2\ e^{[ (\rho \ t +x)\ \sqrt{\tau _2}]}+\tau _1 \left( 2 \ \tau _2-1\right) \right) }{\tau _1-2 \tau _2 \ e^{[(x+\rho \ t)\ \sqrt{\tau _2}] }}\right] e^{i (- x \kappa +\zeta + t \omega )}. \end{aligned}$$

## Summary of solution types and physical interpretations

This section provides a comprehensive summary of the diverse exact solutions obtained for the coupled nonlocal Lakshmanan-Porsezian-Daniel (LPD) equations through the extended F-expansion method. The rich variety of solution types presented below demonstrates the complex nonlinear dynamics governed by the system. Table 1 categorizes these solutions, their mathematical forms, key parameter constraints, and detailed physical interpretations in the context of wave propagation in nonlocal nonlinear media.

**Table 1:** Summary of exact solution types for the coupled nonlocal LPD equations.

Solution type	Reference	Mathematical form (*G* component)	Physical interpretation
Bright Soliton	Eqs.(19), and (20)	$\sqrt{\frac{1}{h^2+1}} \ {{\,\textrm{sech}\,}}\left( x-4 \kappa ^3 t\right) ~ e^{i (- x \kappa +\zeta + t \omega )}$	Represents a stable, localized wave packet that maintains its shape and velocity upon propagation. Models optical pulse transmission in defocusing nonlinear media with nonlocal response.
Singular Periodic	Eqs.(21), and (22)	$2 \ \sqrt{\frac{3}{7 \left( h^2+1\right) }} \csc \left[ x+\left( \frac{13 \kappa }{6}-4 \kappa ^3\right) t\right] ~ e^{i (- x \kappa +\zeta + t \omega )}$	Describes periodic wave patterns with singularities at regular intervals. Physically represents wave collapse phenomena in periodic structures and diffraction-managed systems.
Singular Soliton	Eqs.(25), and (26)	$2 \ \sqrt{\frac{3}{7 \left( h^2+1\right) }} {{\,\textrm{csch}\,}}\left[ x-\left( \frac{13 \kappa }{6}+4 \kappa ^3\right) t\right] ~ e^{i (- x \kappa +\zeta + t \omega )}$	Corresponds to localized waves with singularities, modeling rogue wave behavior and extreme wave events in nonlocal media with specific parameter conditions.
Hyperbolic Solution	Eqs.(27), and (28)	$2 \ \sqrt{\frac{3}{7 \left( h^2+1\right) }} \sinh \left[ x-\left( \frac{13 \kappa }{6}+4 \kappa ^3\right) t\right] ~ e^{i (- x \kappa +\zeta + t \omega )}$	Exhibits unbounded exponential growth, representing modulation instability and wave amplification processes in nonlinear systems with gain mechanisms.
Rational Solution	Eqs.(31), and (32)	$-\frac{2\ \alpha \ \sqrt{3}}{(x+\rho \ t)\sqrt{7\ (h^2+1)}} \ e^{i(-\kappa x+\zeta +\omega t)}$	Algebraic solution that decays algebraically in both time and space. Models dispersive wave radiation and background oscillations in nonlinear wave systems.
Jacobi Elliptic	Eqs.(33), and (34)	$\small {\sqrt{\frac{m^2}{h^2+1}}{{\,\textrm{cn}\,}}\left[ x+(\alpha ^2\kappa (m^2-1)-4\kappa ^3)t\right] e^{i(-\kappa x+\zeta +\omega t)}}$	General periodic solution that interpolates between sinusoidal waves ($m\rightarrow 0$) and solitons ($m\rightarrow 1$). Represents nonlinear wave trains in periodic potentials.
Dark Soliton	Eqs.(41), and (42)	$\sqrt{-\frac{12 \ \tau _2}{14 \left( h^2+1\right) }} \ \tanh \left[ (\rho \ t +x) \ \sqrt{-\frac{\tau _2}{2}} \right] ~ e^{i (- x \kappa +\zeta + t \omega )}$	Describes intensity dips on a continuous wave background. Models void solitons and dark pulse propagation in focusing nonlinear media with nonlocal response.
Weierstrass Elliptic	Eqs.(57), and (58)	$\pm A(x, t) \ \left[ \frac{2 \left[ 4 \tau _0+\tau _1 \wp \left( \sqrt{\frac{\tau _3}{4}} (\rho t+x),-\frac{4 \tau _1}{\tau _3},-\frac{4 \tau _0}{\tau _3}\right) \right] }{\tau _1 \sqrt{\left( h^2+1\right) \kappa } \ \wp \left( \sqrt{\frac{\tau _3}{4}} (\rho t+x),-\frac{4 \tau _1}{\tau _3},-\frac{4 \tau _0}{\tau _3}\right) }\right]$	The most general doubly periodic solution, representing quasi-periodic wave patterns in anisotropic nonlinear media.
Exponential Solution	Eqs.(59), and (60)	$B(x, t)\ \left[ \frac{6 \left( 2 \ \tau _2\ e^{[ (\rho \ t +x)\ \sqrt{\tau _2}]}+\tau _1 \left( 2 \ \tau _2-1\right) \right) }{\tau _1-2 \tau _2 \ e^{[(x+\rho \ t)\ \sqrt{\tau _2}] }}\right]$	Non-periodic solution showing pure exponential behavior, modeling wave amplification/attenuation and boundary layer effects.

where $$A(x, t)=\frac{\sqrt{3 \left( 4 \kappa ^3+\rho \right) }}{\sqrt{91 \left( h^2+1\right) \kappa }}~ \ e^{i (- x \kappa +\zeta + t \omega )}, \ \text {and} \ B(x, t)= - \frac{\sqrt{\left( 4 \kappa ^3+\rho \right) }}{\sqrt{91 \left( h^2+1\right) \kappa }}~ e^{i (- x \kappa +\zeta + t \omega )}.$$

## Modulation instability

Modulation instability (MI), a well-studied phenomenon in nonlinear physics, refers to the exponential growth of minor perturbations superimposed on a continuous wave (CW) solution in nonlinear systems. This often leads to the formation of localized structures, such as pulse trains, and is significant across various fields, including hydrodynamics, engineering, fiber optics, and plasma physics. When nonlinear and dispersive effects interact, many nonlinear processes exhibit instability in steady-state modulation. In the context of optical fibers, where it is crucial for designing communication systems, modulation instability has been extensively researched, with nonlinearity arising from the optical signal traveling through the fiber’s wave field.

The main goal of this section is to analyze the equation’s MI using the linear stability technique.

We consider that the system under study has the steady-state solutions as follows:

63 $$\begin{aligned} G =\left[ \mathcal {F}(x,t)+\sqrt{\mathcal {A}}\right] \ e^{i \mathcal {A} t}, \end{aligned}$$

64 $$\begin{aligned} Q =\left[ \mathcal {H}(x,t)+\sqrt{\mathcal {A}} \right] \ e^{i \mathcal {A} t}, \end{aligned}$$

The normalized power value is denoted by $\mathcal {A}$, with disturbance terms represented by $\mathcal {F}(x,t)$ and $\mathcal {H}(x,t)$. By substituting Eqs. (63) and (64) into Eqs. (1) and (2), and then applying linearization, we obtain:

65 $$\begin{aligned} \mathcal {F}_{\text {xxxx}}+14 \mathcal {A}\ \mathcal {F}_{\text {xx}}+6 \mathcal {A} \ \mathcal {H}_{\text {xx}}+i \mathcal {F}_t+\left( 72 \mathcal {A}^2-\mathcal {A}\right) \left( \mathcal {F}^*+\mathcal {F}\right) +48 \mathcal {A}^2 \left( \mathcal {H}^*+\mathcal {H}\right) =0, \end{aligned}$$

66 $$\begin{aligned} \mathcal {H}_{\text {xxxx}}+14 \mathcal {A}\ \mathcal {H}_{\text {xx}}+6 \mathcal {A} \ \mathcal {F}_{\text {xx}}+i \mathcal {H}_t+\left( 72 \mathcal {A}^2-\mathcal {A}\right) \left( \mathcal {H}^*+\mathcal {H}\right) +48 \mathcal {A}^2 \left( \mathcal {F}^*+\mathcal {F}\right) =0. \end{aligned}$$

However, in mathematical notation, the conjugate of a complex function is represented by the symbol $*$. It is significant to remember that the perturbation of $\mathcal {F}(x, t)$ will be derived. Furthermore, one may similarly obtain the mutual information (MI) analysis associated with the perturbation $\mathcal {H}(x, t)$ by using the same approach that we did for this derivation. By using this method, we establish a clear connection between the perturbations we study and the ensuing MI inquiry, guaranteeing that both facets are fully covered and comprehended.

Presume the following can be used to express the solution for the system in Eqs. (65) and (66):

67 $$\begin{aligned} \mathcal {F} =f_1 \ e^{i (L\ x-\omega \ t)}+f_2 \ e^{-i (L \ x-\omega \ t)}, \end{aligned}$$

and

68 $$\begin{aligned} \mathcal {H} =f_1 \ e^{i (L\ x-\omega \ t)}+f_2 \ e^{-i (L \ x-\omega \ t)}, \end{aligned}$$

where the normalized wave number is *L* and the perturbation frequency is $\omega$.

Substituting Eqs. (67) and (68) into the linearized system (65) and (66) yields expressions that must vanish identically for all values of *x* and *t*. This fundamental requirement implies that the coefficients of the exponentially varying terms $e^{i(L x - \omega t)}$ and $e^{-i(L x - \omega t)}$ must separately equal zero.

The condition of vanishing coefficients leads to a homogeneous system of equations for the amplitudes $f_1$ and $f_2$. For non-trivial solutions ($f_1, f_2 \ne 0$) to exist, the determinant of the system’s coefficient matrix must vanish. This solvability condition yields the characteristic equation:

$$\det \begin{pmatrix} A_1 & A_2 \\ B_1 & B_2 \\ \end{pmatrix} = 0,$$

where the matrix elements $A_1, A_2, B_1, B_2$ are functions of $\omega$, *L*, and $\mathcal {A}$. Solving this determinant condition produces the dispersion relation:

69 $$\begin{aligned} \omega =\pm L \ \sqrt{\left( L^2-20 \ \mathcal {A}\right) \left( 2 \ \mathcal {A} (120\ \mathcal {A}-1)+L^4-20\ \mathcal {A} \ L^2\right) }. \end{aligned}$$

Equation (69) provides a solid foundation for understanding the linear stability analysis of the system’s steady-state conditions. A real value for the parameter $\omega$ indicates stability, meaning the system can maintain its steady-state response over time, even with minor perturbations. Conversely, an imaginary value of $\omega$ signifies instability, where disturbances will exponentially grow, leading to diverging behavior. To assess this instability, the following techniques are employed to develop and determine the modulation instability gain spectrum, which quantifies the level of instability.

70 $$\begin{aligned} G (\mathcal {A})=2 ~ \text {Im}\left[ \pm L \ \sqrt{\left( L^2-20 \ \mathcal {A}\right) \left( 2 \ \mathcal {A} (120\ \mathcal {A}-1)+L^4-20\ \mathcal {A} \ L^2\right) }\right] . \end{aligned}$$

Figure 5 presents a detailed three-dimensional analysis of the gain spectrum $|G(\mathcal {A}, L)|$ as a function of both wave number *L* and normalized power $\mathcal {A}$. This representation clearly demonstrates how the instability regions evolve across the parameter space, showing the expansion of unstable bands with increasing power levels. The plot reveals maximum gain values reaching approximately 20,000 at specific parameter combinations, indicating highly unstable regions where perturbations experience significant exponential growth. Also, this figure provides a complementary two-dimensional cross-sectional view of the gain spectrum at $\mathcal {A} = 5.2$, showing the precise relationship between wave number *L* and gain magnitude |*G*|. The asymmetric shape of the gain spectrum indicates different growth rates for various perturbation frequencies, with a rapid increase followed by a more gradual decrease in gain magnitude. These additional graphical representations enhance our understanding of the system’s stability characteristics and provide valuable insights for practical applications where controlling modulation instability is crucial, such as in optical communication systems and nonlinear wave propagation environments. The comprehensive graphical analysis confirms the theoretical predictions and offers complete characterization of the instability properties across the parameter space, particularly highlighting the dangerous parameter regions where extreme instability occurs.

## Graphic representation of solutions

**Figure 1:**
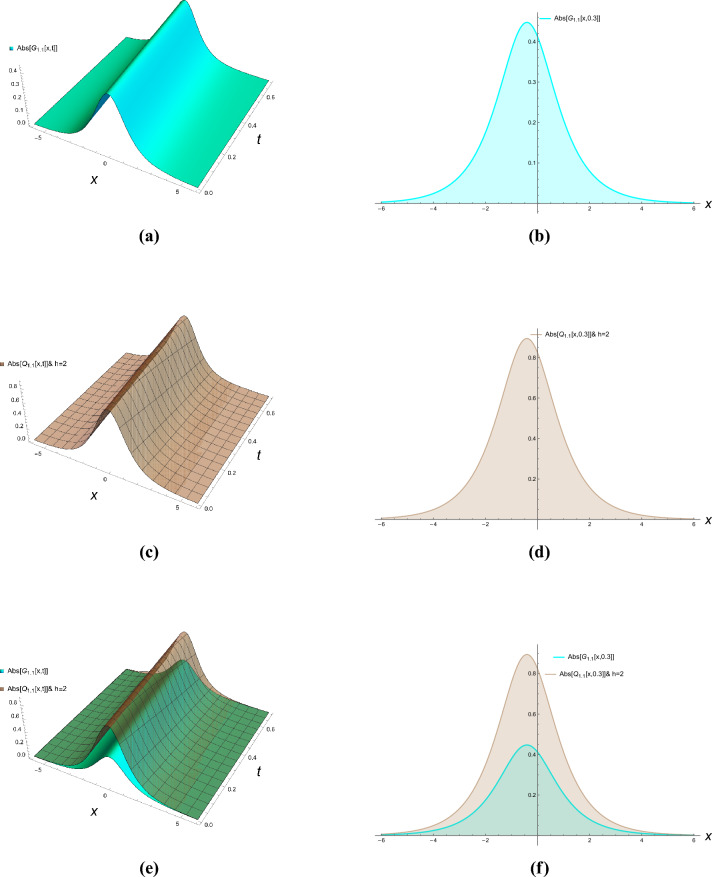
Bright solitons of Eqs. (21) and (22).

**Figure 2:**
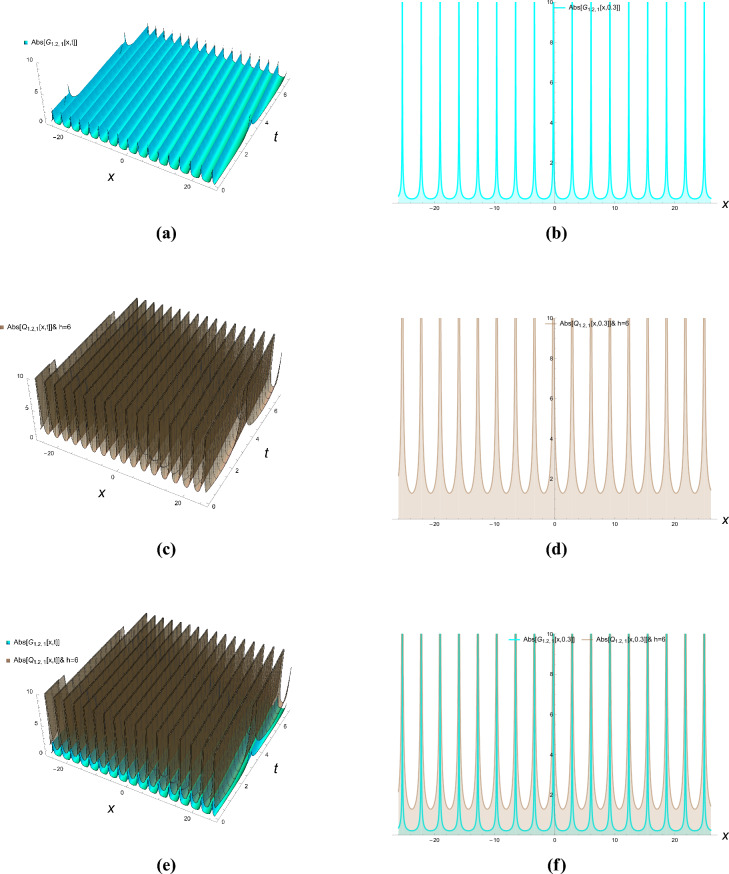
Singular periodic solutions of Eqs. (23) and (24).

**Figure 3:**
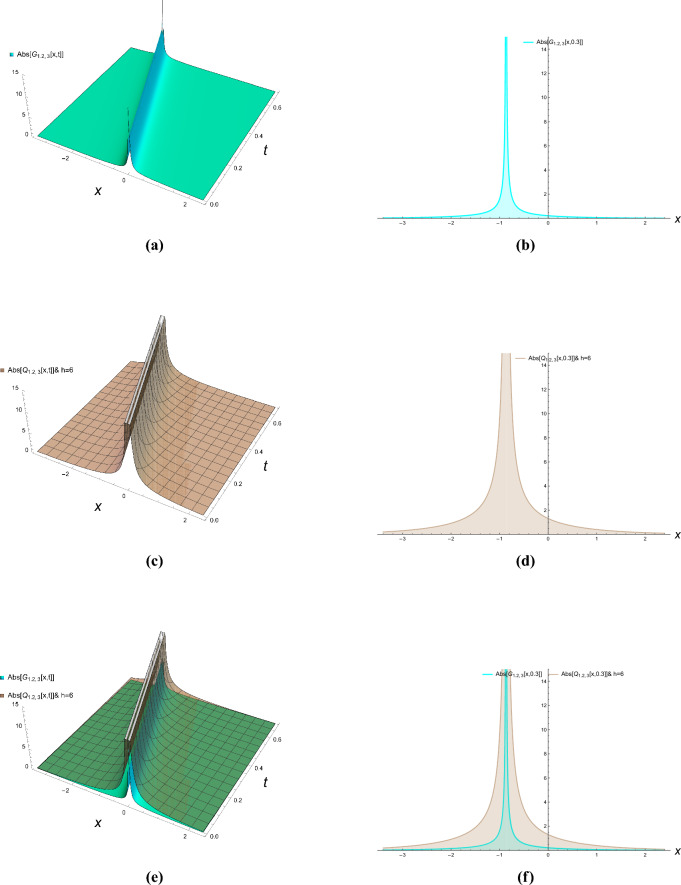
Singular solitons of Eqs. (27) and (28).

**Figure 4:**
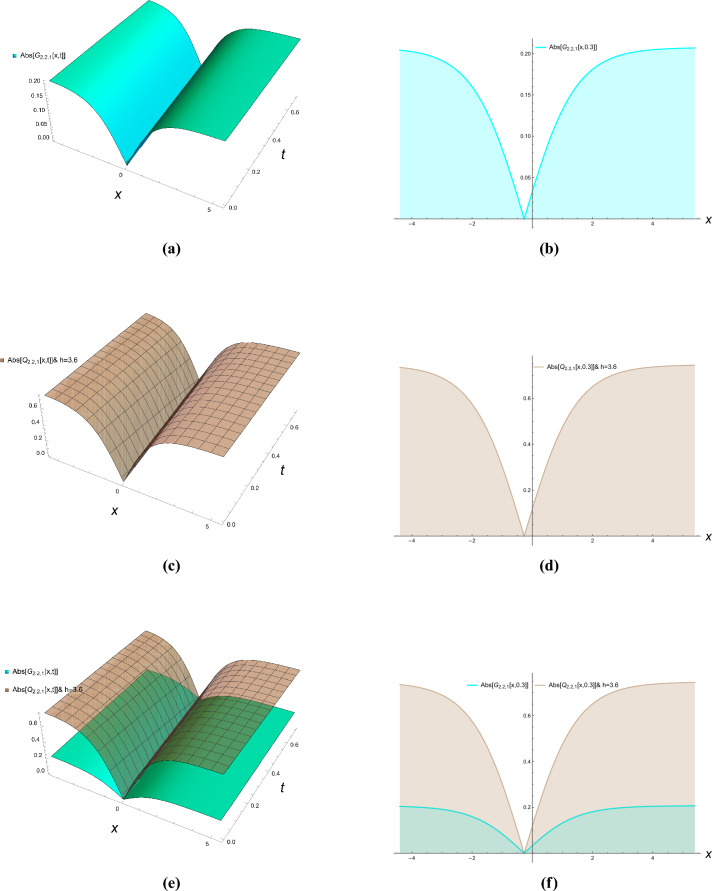
Dark solitons of Eqs. (43) and (44).

This section presents comprehensive graphical simulations of the obtained solutions to elucidate their physical characteristics and dynamical behaviors. Each figure demonstrates distinct types of soliton solutions extracted from the governing equations, with specific parameter selections to highlight their unique properties. The physical interpretations of these solutions are discussed in the context of wave propagation in nonlinear dispersive media. Figure 1 illustrates the bright soliton solution described by Eqs. (21) and (22) with parameter values $\kappa = -0.7$, $\zeta = 0.8$, and $h = 2$. Bright solitons, characterized by a localized intensity peak on a zero background, represent stable wave packets that maintain their shape during propagation through a balance between nonlinearity and dispersion. These solutions are particularly significant in optical fibers and Bose-Einstein condensates, where they describe self-trapped waves that propagate without distortion. The figure clearly demonstrates the characteristic sech-shaped profile with amplitude modulation along the temporal dimension. The singular periodic solutions governed by Eqs. (23) and (24) are presented in Fig. 2 for parameter values $\kappa = -0.87$, $\zeta = 0.8$, and $h = 6$. These solutions exhibit periodic singularities with undefined points at regular intervals, representing wave collapse phenomena in certain nonlinear media. Physically, such solutions may describe extreme wave events or breakdown regions in plasma physics and nonlinear optics where the wave amplitude becomes theoretically infinite at discrete points. The figure shows the periodic repetition of singular points along the propagation direction. Figure 3 displays the singular soliton solution obtained from Equations (27) and (28) with parameters $\kappa = -0.7$, $\zeta = 0.78$, and $h = 6$. Singular solitons represent localized waves with a point singularity, typically manifesting as a dip or cusp in the wave profile. These solutions are physically relevant in describing rogue waves or critical phenomena in nonlinear systems where energy concentration leads to singular behavior. The graphical representation clearly shows the localized nature of the singularity and its spatial confinement. The dark soliton solution characterized by Equations (43) and (44) is depicted in Fig. 4 for parameter values $\kappa = -0.8$, $\rho = 0.9$, $\omega = 0.8$, $\tau _2 = -0.7$, $\zeta = 0.78$, and $h = 3.6$. Dark solitons appear as intensity dips on a continuous wave background and represent regions of phase discontinuity. These solutions are physically significant in describing void-like structures in nonlinear media and have been experimentally observed in optical fibers and matter waves. The figure demonstrates the characteristic tanh-shaped profile with a phase jump across the soliton center. The three-dimensional plots show the evolution of wave profiles along both spatial and temporal dimensions, while the contour plots provide complementary information about intensity distribution and gradient variations. The careful selection of parameter values enables the demonstration of both qualitative features and quantitative relationships between various soliton types. These graphical representations not only validate the analytical solutions but also provide physical insights into the behavior of nonlinear waves in the considered system. The distinct characteristics of each solution type–whether bright, dark, or singular–highlight the rich dynamical diversity achievable in nonlinear dispersive media under appropriate conditions.

**Figure 5:**
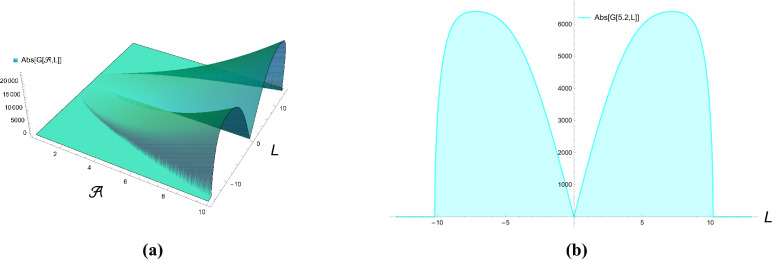
MI gain spectrum for Eq. (70).

## Conclusion

This study primarily focused on the coupled system of the new nonlocal LPDE. The Extended F-Expansion method was first applied to explore the solitons and other solutions of proposed model. Our analysis produced a range of solutions, such as bright, dark, and singular solitons, as well as singular periodic wave solutions, periodic wave solutions, Jacobi elliptic wave solutions, exponential wave solutions, hyperbolic wave solutions, and Weierstrass elliptic solutions. We ensure the presence of the obtained soliton solutions by using the constraints in the parameters. Graphic representations of such solutions were also introduced as a way to clarify the physical nature of some solutions. The solutions extracted in our paper are novel, and this model hasn’t studied using this technique before.

The investigation of MI indicates crucial insights into the solutions’ stability. A detailed analysis was performed to determine the conditions under which MI occurs, demonstrating that certain parameters within the system can lead to instabilities that enhance wave amplitude and alter wave shapes dynamically. These results contribute significantly to the theoretical understanding of nonlinear wave propagation and can potentially influence experimental designs in related fields. The findings are pertinent in various physical phenomena wherein LPD model real-world applications, including optical fibers, plasmas, and hydrodynamics. The ability to predict and control MI in these contexts opens new avenues for developing advanced materials and technologies, particularly in optics and energy transmission systems. Future research should focus on extending these methods to higher-dimensional cases and examining their applicability in more complex nonlinear systems. Moreover, exploring numerical simulations to validate the analytical solutions will be a critical step in bridging theoretical predictions with experimental results, fostering a more comprehensive understanding of dynamic instabilities in various media.

## Data Availability

The datasets used and/or analyzed during the current study are available from the corresponding author upon reasonable request.
